# Supplementary material for: Differential methylation analysis in neuropathologically confirmed dementia with Lewy bodies
Source: Commun Biol. 2024 Jan 5;7:35. doi: 10.1038/s42003-023-05725-x (PMC10770032; doi:10.1038/s42003-023-05725-x)
Supplement: Supplementary file 2 — Supplementary Information [file 42003_2023_5725_MOESM2_ESM.pdf]

Differential methylation analysis in neuropathologically confirmed dementia with Lewy bodies

Paolo Reho et al.

Contents

Supplementary Figures	Page
**Supplementary Figure 1.** Volcano plot, QQ-plot, and Manhattan plot of the EWAS using *bacon*	2
**Supplementary Figure 2.** Expression plot of the differentially methylated probes	3
**Supplementary Figure 3.** Correlation plot of DNA methylation principal components and covariates	4
**Supplementary Figure 4.** QQ-plot and Manhattan plot of the EWAS using OSCA-MOA	5
**Supplementary Figure 5.** Comparison of *limma* and OSCA-MOA EWAS	6
**Supplementary Figure 6.** Age and sex distribution of DLB cases and controls used in the EWAS analysis	7
**Supplementary Figure 7.** Quality control analyses	8
**Supplementary Figure 8.** *ß*-values distribution in the DLB EWAS data	9
**Supplementary Figure 9.** Z-scores comparison between beta- and M-values-based EWAS	10
**Supplementary Tables/Data**	
**Supplementary Table 1.** Demographic characteristics of study participants	11

Supplementary Figure 1. Volcano plot, QQ-plot, and Manhattan plot of the EWAS using *bacon*

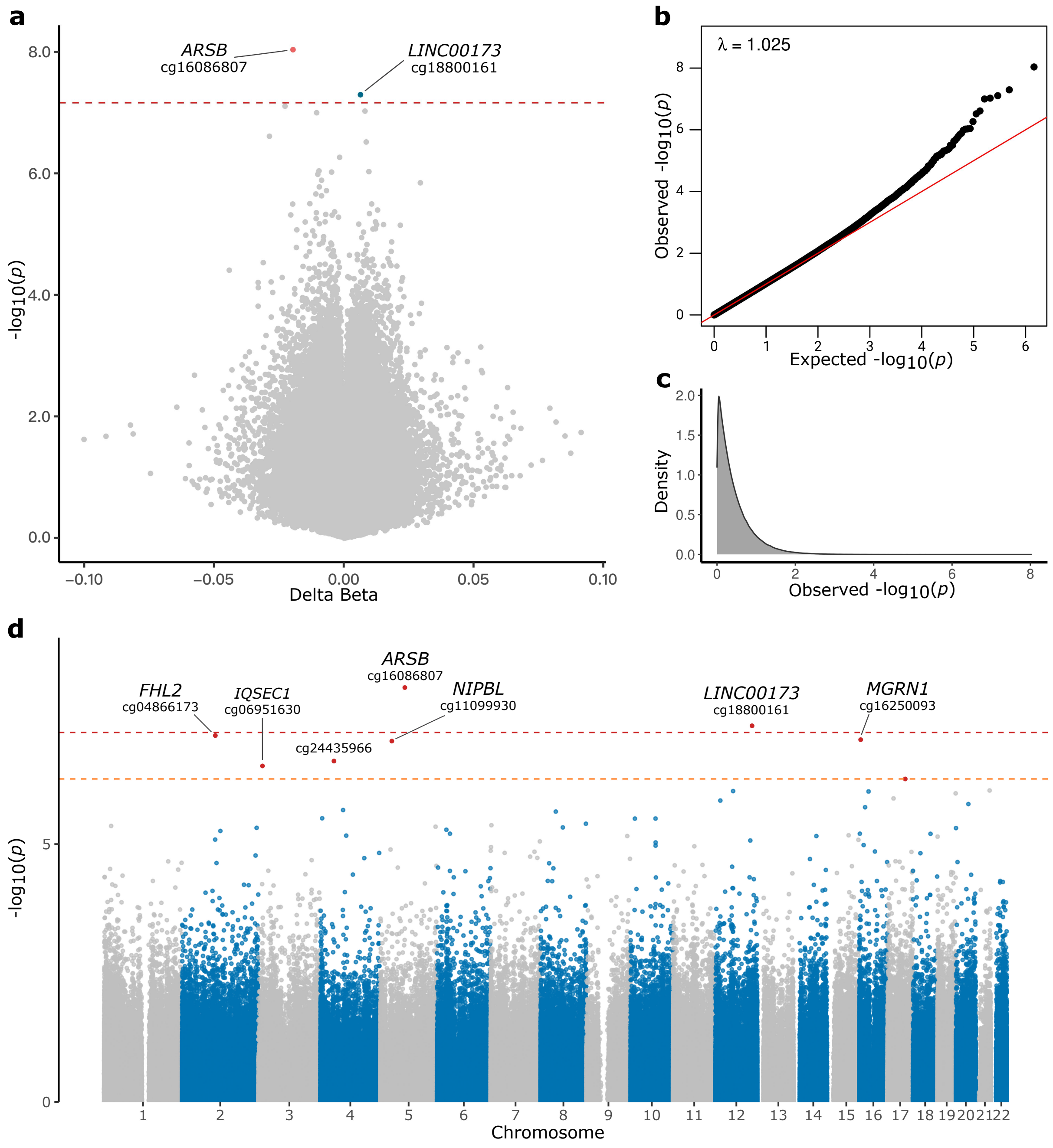

Volcano plot **a)** showing statistical significance (-log10 *p*-value) and magnitude of change (Δβ) of all CpG sites included in the EWAS. Red dots indicate significantly hypomethylated DMPs, and hypermethylated CpGs are displayed as blue dots. The Bonferroni adjusted *p*-value < 0.05 threshold is shown as a red dashed line. QQ-plot **b)** showing the *p*-value distribution and inflation (lambda values, top left). Density plot **c)** illustrating the observed p-values distribution. Manhattan plot **d)** demonstrating the *p*-values of the probes across the genome. The Bonferroni adjusted *p*-value < 0.05 threshold is shown as a red dashed line, while the orange dashed line represents the FDR-significance threshold. Probes surpassing the genome-wide significance are shown as red dots. *P*-values have been corrected using the Bioconductor package *bacon*.

Supplementary Figure 2. Expression plot of the differentially methylated genes

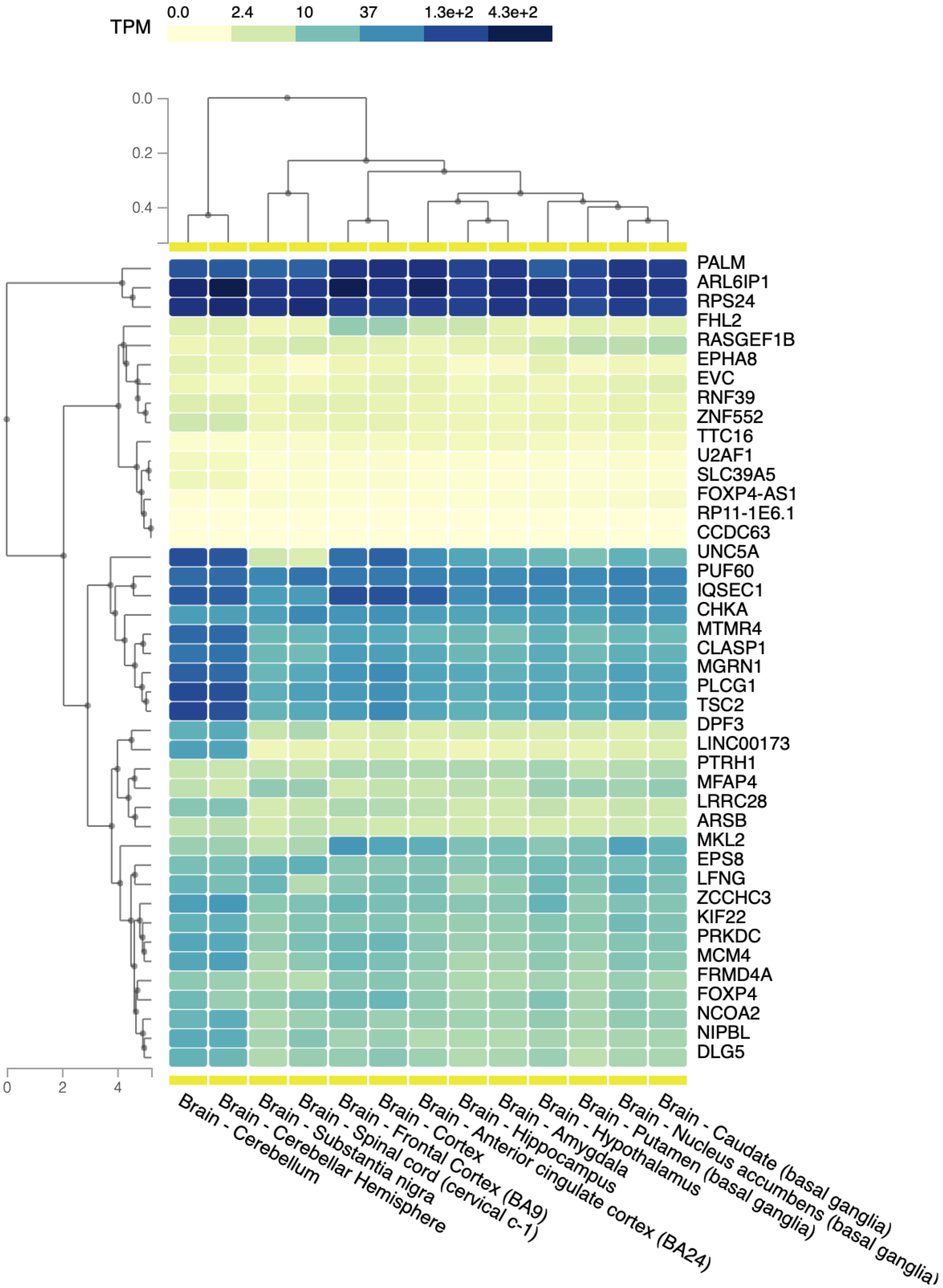

Heatmap plot showing the expression pattern of the differentially methylated genes in different brain regions obtained from Genotype-Tissue Expression (GTEx) database (https://www.gtexportal.org). Light yellow boxes show low expression genes while dark blue boxes identify highly expressed genes. Abbreviation: Transcripts Per Million, TPM.

Supplementary Figure 3. Correlation plot of DNA methylation principal components and covariates

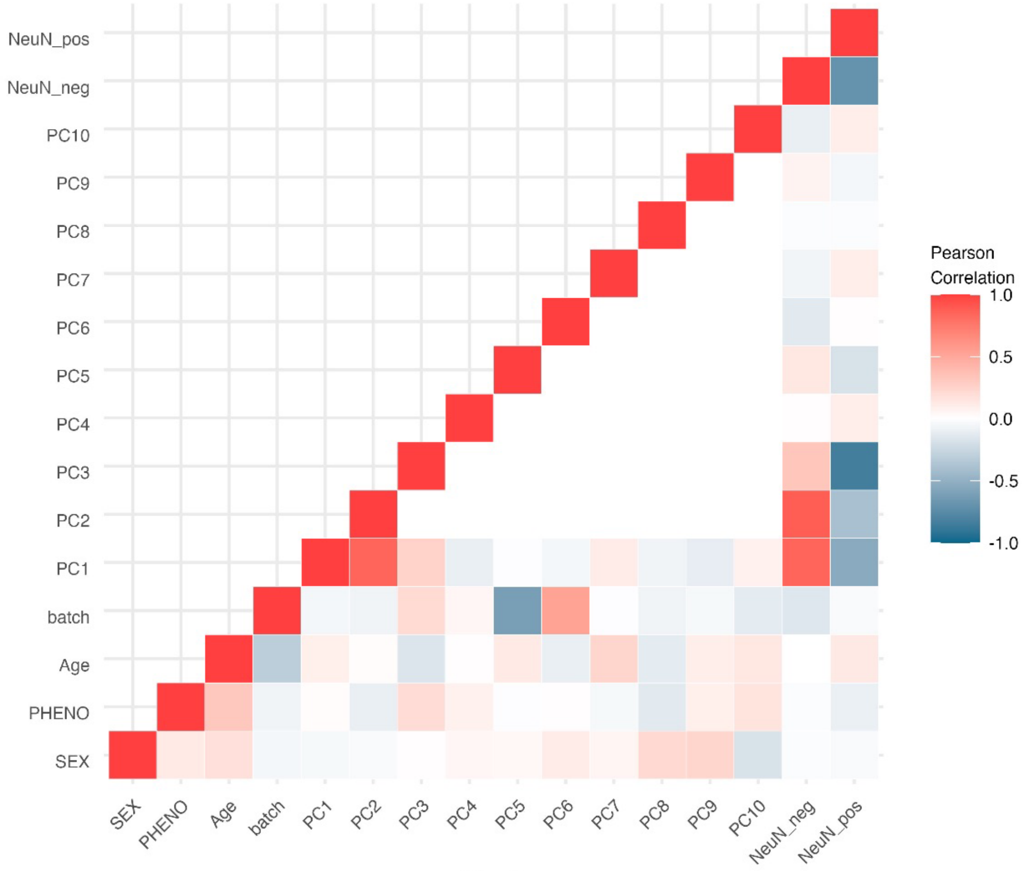

Correlation plot showing the Pearson correlation matrix between ten principal components derived from DNA methylation data, sex, case-control status (PHENO), age, experimental batch as well as cell type proportion (NeuN_neg, neuronal-specific protein-negative ; NeuN_pos, neuronal-specific protein-positive).

Supplementary Figure 4. QQ-plot and Manhattan plot of the EWAS using OSCA-MOA

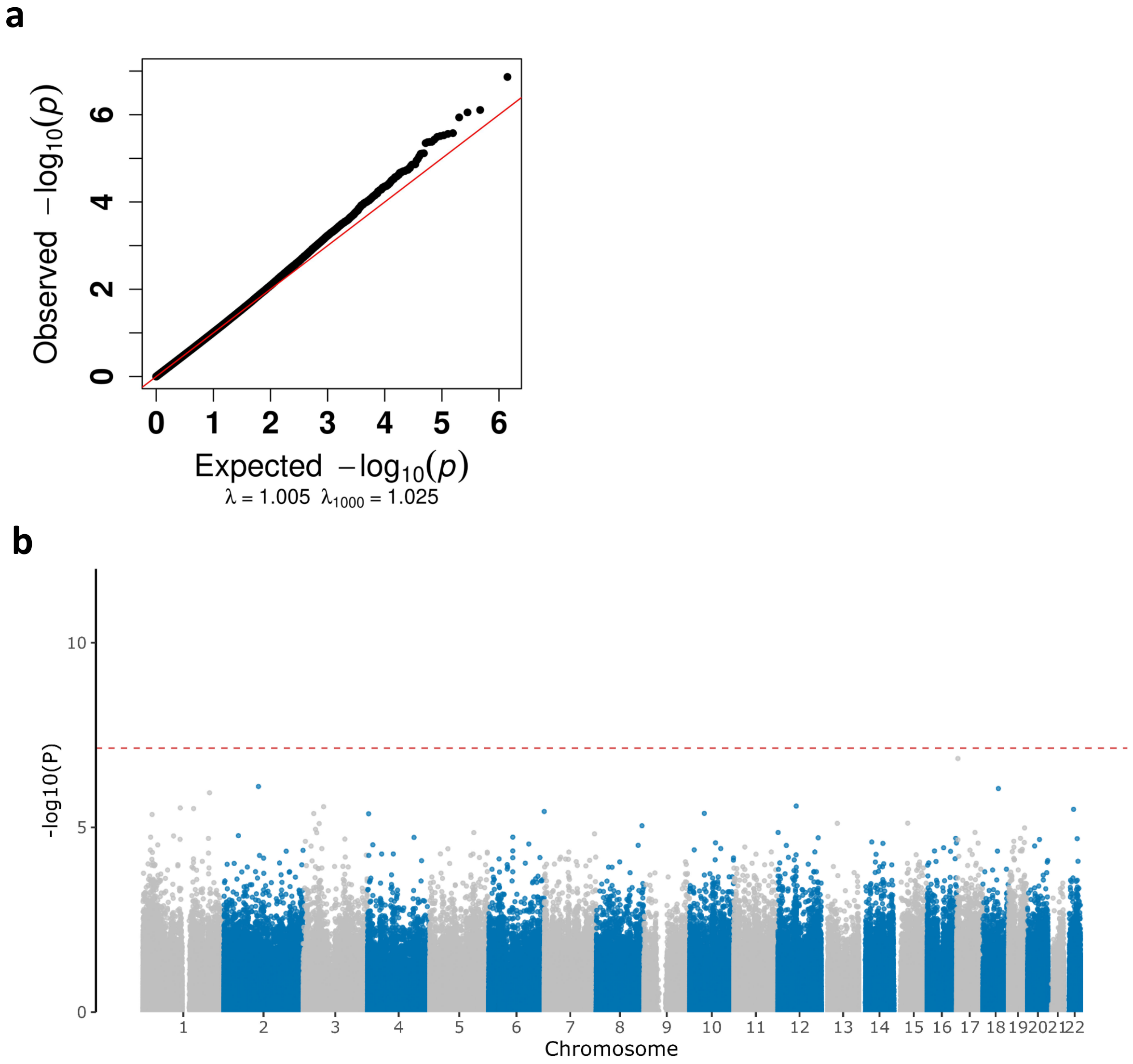

QQ-plot **a)** showing the *p*-value distribution and inflation. Manhattan plot **b)** demonstrating the *p*-values of the probes across the genome. The Bonferroni adjusted *p*-value < 0.05 threshold is shown as a red dashed line. *P*-values have been corrected using the OSCA-MOA tool.

Supplementary Figure 5. Comparison of the *limma* and OSCA-MOA EWAS

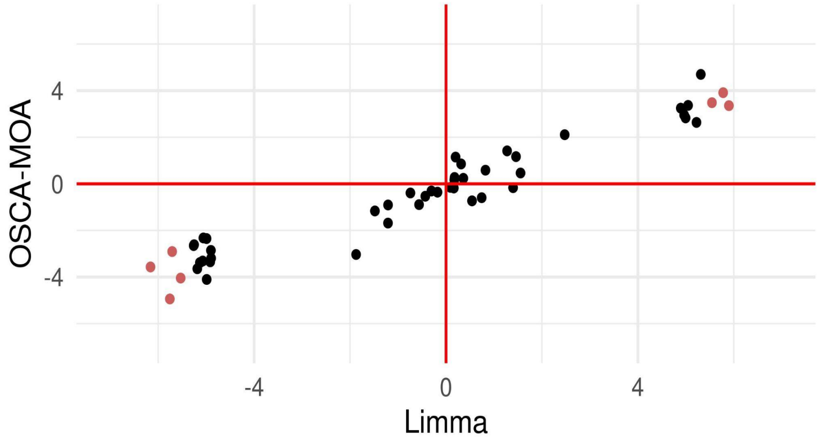

Comparison between top 48 CpG sites (FDR significant probes) obtained from OSCA-MOA and Limma approaches. Data points were transformed as log-fold change divided by the standard error. Red dots show the top seven probes that surpassed the Bonferroni threshold for genome-wide significance.

Supplementary Figure 6. Age and sex distribution of DLB cases and controls used in the EWAS analysis

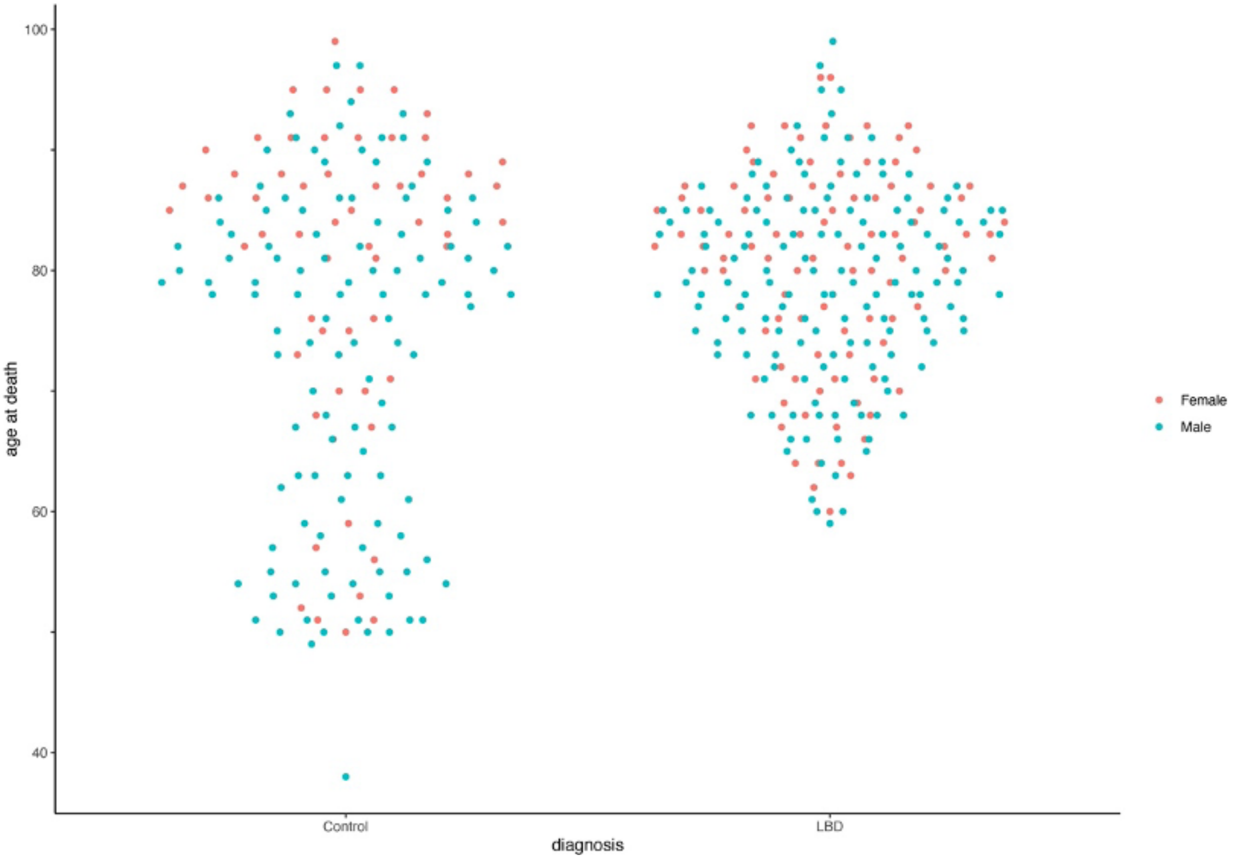

Scatter plot comparing the age and sex distribution of the DLB cases and controls in the study cohort.

Supplementary Figure 7. Quality control analyses

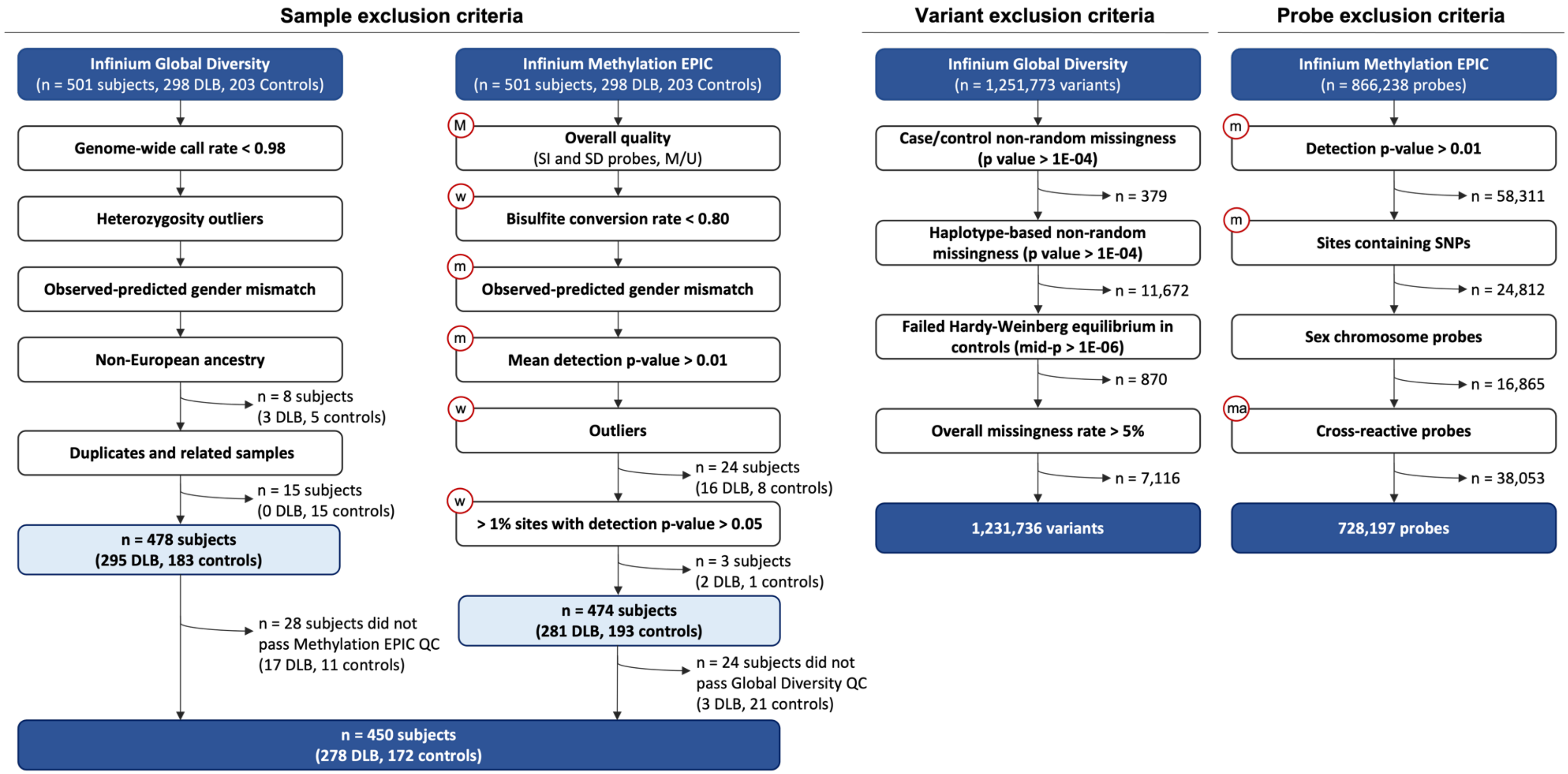

Flow diagrams of sample-level and probe-level quality control steps for the Illumina Epic methylation data in pathologically confirmed DLB cases and controls. Raw signal intensity files (.idat files) were imported into R and processed using the *MethylAid* (v.1.24.0), *minfi* (v.1.36.0), *wateRmelon* (v.1.34.0), and *maxprobes* (v.0.0.2) packages. The R packages used in each step are shown in the red circles: MethylAid (M), minfi (m), wateRmelon (w), and maxprobes (ma). Abbreviations: SI, sample-independent; SD, sample-dependent; M/U, methylated/unmethylated probes ratio; SNPs, single nucleotide polymorphisms.

Supplementary Figure 8. *ß*-values distribution in the DLB EWAS data

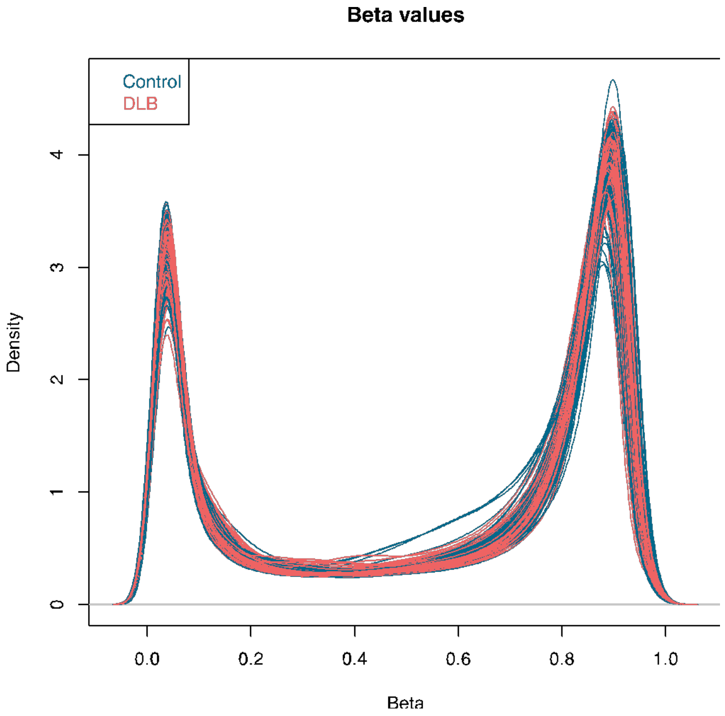

Density plot showing the distribution of normalized DNA methylation levels, as measured by *ß*-values, for 728,197 CpGs evaluated in the DLB EWAS (*n* = 278 DLB cases [blue lines] and 172 controls [red lines]). *ß*-values of 0 indicated unmethylated probes and *ß*-values values of 1 indicate methylated probes.

Supplementary Figure 9. Z-scores comparison between beta- and M-values-based EWAS

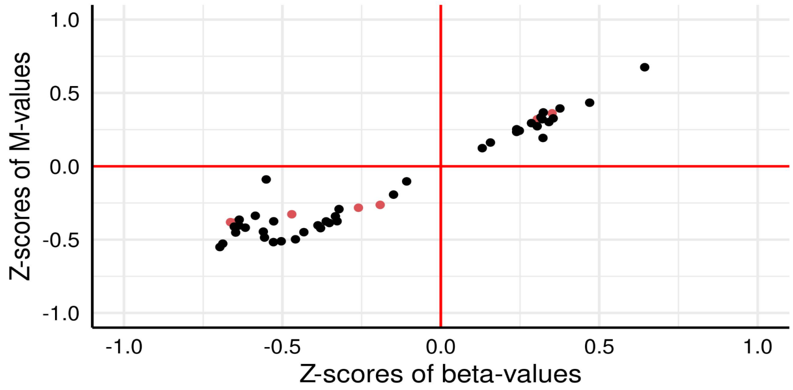

Z-scores comparison between top 48 CpG sites (FDR-significant probes) obtained using M- and beta-values. Z-scores were computed as delta beta (or M) / standard deviation. Red dots show the top seven significant probes that exceeded the Bonferroni threshold.

Supplementary Table 1. Demographic characteristics of study participants

Diagnosis	Sex	Mean Age at Death (Range)	N	Total
Control	M	67.5 yrs (38-97)	134 (66.0%)	203
	F	74.5 yrs (50-99)	69 (34.0%)	
DLB	M	78 yrs (57-99)	177 (59.4%)	298
	F	78 yrs (60-99)	121 (40.6%)	
