# Supplementary material for: Differential methylation analysis in neuropathologically confirmed dementia with Lewy bodies
Source: Commun Biol. 2024 Jan 5;7:35. doi: 10.1038/s42003-023-05725-x (PMC10770032; doi:10.1038/s42003-023-05725-x)
Supplement: Supplementary file 3 — Description of Additional Supplementary Files [file 42003_2023_5725_MOESM3_ESM.docx]

**Description of Additional Supplementary Files**

**Supplementary Data 1.** Differentially methylated probes in DLB

**Supplementary Data 2.** Differentially methylated probes in the sex-specific EWAS

**Supplementary Data 3.** Differentially methylated probes and regions previously associated with DLB, Parkinson’s disease and Alzheimer’s disease

**Supplementary Data 4.** Source data for Figures 2 and 4
