# Supplementary material for: Differential methylation analysis in neuropathologically confirmed dementia with Lewy bodies
Source: Commun Biol. 2024 Jan 5;7:35. doi: 10.1038/s42003-023-05725-x (PMC10770032; doi:10.1038/s42003-023-05725-x)
Supplement: Supplementary file 8 — Reporting summary [file 42003_2023_5725_MOESM8_ESM.pdf]

nature portfolio

Corresponding author(s): Sonja W. Scholz

Last updated by author(s): Dec 12, 2023

Reporting Summary

Nature Portfolio wishes to improve the reproducibility of the work that we publish. This form provides structure for consistency and transparency in reporting. For further information on Nature Portfolio policies, see our Editorial Policies and the Editorial Policy Checklist.

Statistics

For all statistical analyses, confirm that the following items are present in the figure legend, table legend, main text, or Methods section.

n/a	Confirmed	
☐	☒	The exact sample size (n) for each experimental group/condition, given as a discrete number and unit of measurement
☐	☒	A statement on whether measurements were taken from distinct samples or whether the same sample was measured repeatedly
☐	☒	The statistical test(s) used AND whether they are one- or two-sided<br>*Only common tests should be described solely by name; describe more complex techniques in the Methods section.*
☐	☒	A description of all covariates tested
☐	☒	A description of any assumptions or corrections, such as tests of normality and adjustment for multiple comparisons
☐	☒	A full description of the statistical parameters including central tendency (e.g. means) or other basic estimates (e.g. regression coefficient) AND variation (e.g. standard deviation) or associated estimates of uncertainty (e.g. confidence intervals)
☐	☒	For null hypothesis testing, the test statistic (e.g. F, t, r) with confidence intervals, effect sizes, degrees of freedom and P value noted<br>*Give P values as exact values whenever suitable.*
☒	☐	For Bayesian analysis, information on the choice of priors and Markov chain Monte Carlo settings
☒	☐	For hierarchical and complex designs, identification of the appropriate level for tests and full reporting of outcomes
☐	☒	Estimates of effect sizes (e.g. Cohen's d, Pearson's r), indicating how they were calculated

Our web collection on statistics for biologists contains articles on many of the points above.

Software and code

Policy information about availability of computer code

Data collection	Analyses were performed using open-source tools, and the code for the analysis is available at the associated website of each software package.
Data analysis	Specific software packages and versions have been described in the Methods section. The analysis code has been published on Github: https://github.com/pireho/EWAS-Lewy_body_dementia

For manuscripts utilizing custom algorithms or software that are central to the research but not yet described in published literature, software must be made available to editors and reviewers. We strongly encourage code deposition in a community repository (e.g. GitHub). See the Nature Portfolio guidelines for submitting code & software for further information.

Data

Policy information about availability of data

All manuscripts must include a data availability statement. This statement should provide the following information, where applicable:

- Accession codes, unique identifiers, or web links for publicly available datasets
- A description of any restrictions on data availability
- For clinical datasets or third party data, please ensure that the statement adheres to our policy

The EWAS summary statistics have been deposited into the EWAS catalog (www.ewascatalog.org). Individual-level methylation array data are available on dbGaP (accession #: phs001963).

Human research participants

Policy information about studies involving human research participants and Sex and Gender in Research.

Reporting on sex and gender	Sex-bases analyses were performed in this study.
Population characteristics	We used the following covariates for the EWAS analysis: age, sex, experimental batch, and the first five principal components (generated from genotype data using the Infinium Global Diversity Array + Neuro Booster).
Recruitment	Cerebellar brain tissue samples from European-ancestry individuals with a pathological diagnosis of dementia with Lewy bodies were received from North American and European brain donation programs.
Ethics oversight	Our research operates under a research exemption. Brain tissue from autopsies is designated as non-human subjects research according to the revised common rule.

Note that full information on the approval of the study protocol must also be provided in the manuscript.

Field-specific reporting

Please select the one below that is the best fit for your research. If you are not sure, read the appropriate sections before making your selection.

☒ Life sciences ☐ Behavioural & social sciences ☐ Ecological, evolutionary & environmental sciences

For a reference copy of the document with all sections, see nature.com/documents/nr-reporting-summary-flat.pdf

Life sciences study design

All studies must disclose on these points even when the disclosure is negative.

Sample size	Samples from 278 pathologically diagnosed cases diagnosed with dementia with Lewy bodies and 203 neurologically healthy controls were obtained from brain donation programs. All specimens were from European-ancestry individuals.
Data exclusions	Samples without age, sex and diagnosis information were not eligible for inclusion.
Replication	This study does not contain a replication cohort. We highlight this limitation in the Discussion of our manuscript.
Randomization	N/A
Blinding	N/A

Reporting for specific materials, systems and methods

We require information from authors about some types of materials, experimental systems and methods used in many studies. Here, indicate whether each material, system or method listed is relevant to your study. If you are not sure if a list item applies to your research, read the appropriate section before selecting a response.

Materials & experimental systems

n/a	Involved in the study	
☒	☐	Antibodies
☒	☐	Eukaryotic cell lines
☒	☐	Palaeontology and archaeology
☒	☐	Animals and other organisms
☒	☐	Clinical data
☒	☐	Dual use research of concern

Methods

n/a	Involved in the study	
☒	☐	ChIP-seq
☒	☐	Flow cytometry
☒	☐	MRI-based neuroimaging
